# Studies of Laboulbeniales on *Myrmica* ants (IV): host-related diversity and thallus distribution patterns of *Rickia wasmannii*

**DOI:** 10.1051/parasite/2019028

**Published:** 2019-05-20

**Authors:** Danny Haelewaters, Peter Boer, Ferenc Báthori, Zoltán Rádai, Ana Sofia P.S. Reboleira, András Tartally, Walter P. Pfliegler, André De Kesel, Oldřich Nedvěd

**Affiliations:** 1 Farlow Reference Library and Herbarium of Cryptogamic Botany, Harvard University 22 Divinity Avenue Cambridge MA 02138 USA; 2 Faculty of Science, University of South Bohemia Branišovská 31 37005 České Budějovice Czech Republic; 3 Department of Botany and Plant Pathology, Purdue University 915 W. State Street West Lafayette IN 47907 USA; 4 Gemene Bos 12 1861 HG Bergen The Netherlands; 5 Department of Evolutionary Zoology and Human Biology, University of Debrecen Egyetem tér 1 4032 Debrecen Hungary; 6 Natural History Museum of Denmark, University of Copenhagen Universitetsparken 15 2100 København Ø Denmark; 7 Department of Molecular Biotechnology and Microbiology, University of Debrecen Egyetem tér 1 4032 Debrecen Hungary; 8 Meise Botanic Garden Nieuwelaan 38 1860 Meise Belgium

**Keywords:** Ant-associated fungi, Laboulbeniomycetes, Molecular evolution, Ribosomal DNA, Thallus density

## Abstract

Fungal species identities are often based on morphological features, but current molecular phylogenetic and other approaches almost always lead to the discovery of multiple species in single morpho-species. According to the morphological species concept, the ant-parasitic fungus *Rickia wasmannii* (Ascomycota, Laboulbeniales) is a single species with pan-European distribution and a wide host range. Since its description, it has been reported from ten species of *Myrmica* (Hymenoptera, Formicidae), of which two belong to the *rubra*-group and the other eight to the phylogenetically distinct *scabrinodis*-group. We found evidence for *R. wasmannii* being a single phylogenetic species using sequence data from two loci. Apparently, the original morphological description (dating back to 1899) represents a single phylogenetic species. Furthermore, the biology and host-parasite interactions of *R. wasmannii* are not likely to be affected by genetic divergence among different populations of the fungus, implying comparability among studies conducted on members of different ant populations. We found no differences in total thallus number on workers between *Myrmica* species, but we did observe differences in the pattern of thallus distribution over the body. The locus of infection is the frontal side of the head in *Myrmica rubra* and *M. sabuleti* whereas in *M. scabrinodis* the locus of infection differs between worker ants from Hungary (gaster tergites) and the Netherlands (frontal head). Possible explanations for these observations are differences among host species and among populations of the same species in (i) how ant workers come into contact with the fungus, (ii) grooming efficacy, and (iii) cuticle surface characteristics.

## Abstract (fr)

L’identification des espèces fongiques est souvent basée sur des caractéristiques morphologiques, mais les approches phylogénétiques moléculaires et autres conduisent presque toujours à la découverte d’espèces multiples dans une seule morpho-espèce. Selon le concept d’espèce morphologique, le champignon parasite de fourmis *Rickia wasmannii* (Ascomycota, Laboulbeniales) est une espèce unique ayant une répartition paneuropéenne et une large gamme d’hôtes. Depuis sa description, il a été signalé chez dix espèces de *Myrmica* (Hymenoptera, Formicidae), dont deux appartiennent au groupe *rubra* et les huit autres au groupe *scabrinodis*, phylogénétiquement distinct. Nous avons trouvé que *R. wasmannii* était une seule espèce phylogénétique en utilisant les données des séquences de deux loci. Apparemment, la description morphologique originale (datant de 1899) représente une seule espèce phylogénétique. De plus, la biologie et les interactions hôte-parasite de *R. wasmannii* ne devraient pas être affectées par une divergence génétique entre différentes populations du champignon, ce qui implique une comparabilité entre les études conduites sur des membres de différentes populations de fourmis. Nous n’avons trouvé aucune différence dans le nombre total de thalles chez les ouvrières entre les espèces de *Myrmica*, mais nous avons observé des différences dans le schéma de distribution des thalles sur le corps. Le locus d’infection est le front de la tête chez *Myrmica rubra* et *M. sabuleti*, alors que chez *M. scabrinodis*, le lieu d’infection diffère entre les ouvrières de Hongrie (tergites abdominaux) et des Pays-Bas (front de la tête). Les explications possibles de ces observations sont les différences entre les espèces hôtes et entre les populations de la même espèce en ce qui concerne (i) le contact des ouvrières avec le champignon, (ii) l’efficacité du toilettage et (iii) les caractéristiques de la surface de la cuticule.

## Introduction

**Figure 1:**
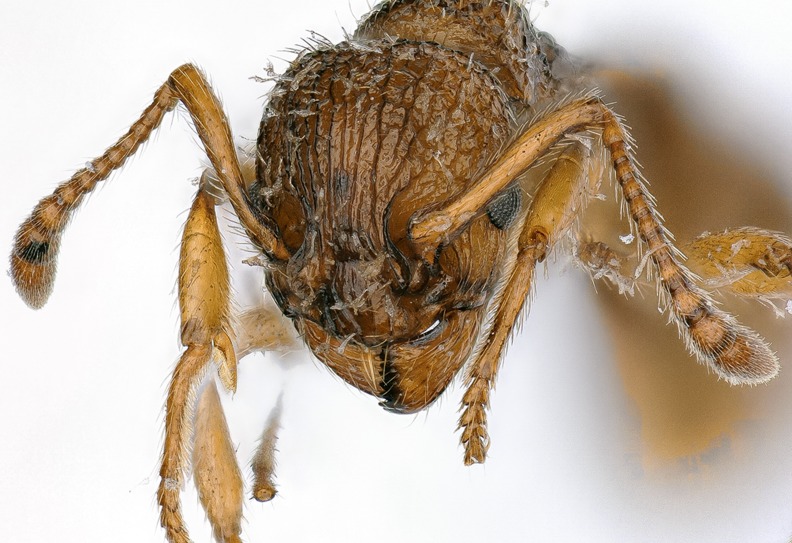
The head of a *Myrmica sabuleti* worker, heavily infected with *Rickia wasmannii*.

Ants harbour a vast diversity of microbial parasites and pathogens. Fungal species of ants are usually pathogenic, but some species, notably members of Laboulbeniales (Ascomycota), are ectoparasitic and do not cause the death of the hosts. Laboulbeniales are developmentally unique in that they do not produce mycelia; instead, they produce multicellular units, *thalli*, which attach externally to the integument of the host. *Rickia wasmannii* Cavara, 1899 [9] (Fig. 1) is a species of Laboulbeniales that infects diverse ants in the genus *Myrmica* Latreille, 1804 (Hymenoptera, Formicidae) in Europe. Knowledge on the biology of *R. wasmannii* is accumulating and this species has quickly become one of the most thoroughly researched species of Laboulbeniales [2–5, 11–13, 15, 29, 32, 52, 53, 70].

Non-random positional patterns on the integument [48], variation in host usage across geographical regions [29], and habitat specificity [53] have recently been explored for *R. wasmannii*. On the other hand, the phylogenetic diversity of *R. wasmannii* from different host species remains unknown. This question deserves to be explored, as it was shown recently for a few Laboulbeniales examples that there is phylogenetic structuring within presumed species. For species in the genera *Gloeandromyces* Thaxt. and *Hesperomyces* Thaxt., phylogenetic segregation by host species has been observed. For example, in both *G. pageanus* Haelew. and *G. streblae* Thaxt., two phylogenetic clades can be found: one clade for isolates removed from *Trichobius dugesioides* Wenzel, 1966 (Diptera, Streblidae) and another clade for isolates from *T. joblingi* Wenzel, 1966 [33, 34]. Similarly, *Hesperomyces virescens* Thaxt. consists of multiple clades, each clade corresponding to a species with strict host specificity [30].

Even though the main hosts of *R. wasmannii* all belong to a single genus of ants (for a discussion of alternative hosts, see [53]), the different host species are placed in two clades that are phylogenetically not closely related (referred to as species groups in [39, 56]). *Myrmica rubra* (Linnaeus, 1758) and *M. ruginodis* Nylander, 1846 belong to the *rubra*-group, whereas the other known hosts belong to the *scabrinodis*-group. These are *M. gallienii* Bondroit, 1920; *M. hellenica* Finzi, 1926; *M. sabuleti* Meinert, 1861; *M. scabrinodis* Nylander, 1846; *M. slovaca* Sadil, 1952; *M. specioides* Bondroit, 1918; *M. spinosior* Santschi, 1931; and *M. vandeli* Bondroit, 1920 [4, 29]. Assessing whether *R. wasmannii* shows phylogenetic segregation by host species or host species group is important to better understand its interactions with different ant hosts. Studies using infected and non-infected *Myrmica* ants have been done to assess the parasite’s effects on ant behaviour and physiology. Interpretation of these results is complicated when the taxonomic status of different fungal populations is uncertain. Comparing interactions between a fungal parasite and its different hosts is only reliable when the fungal populations represent a single phylogenetic species.

Building on the hypothesis that *R. wasmannii* is a complex of species, potentially segregated by host species (or species group), it is logical to assume that thallus distribution patterns may be different on various ant hosts. If we were to find variable patterns of thallus distribution, these would have to be (partly) attributed to the fungal partner, the ant partner, environmental factors, or a combination of these. To try to shed light on this complex interaction of factors, we took an integrative approach (*sensu* [30]) and generated independent sets of data, that is, barcode sequences of *R. wasmannii* isolates and thallus density counts by body part.

During this study, we sampled infected ants from different regions in Europe and sequenced two loci to assess intraspecific phylogenetic diversity in *R. wasmannii*. Collected host ants represent three *Myrmica* species belonging to the *rubra*- and *scabrinodis*-groups [56]. After having accumulated many collections of *R. wasmannii*-infected ants, we assessed thallus densities per body part from different host species (*M. rubra*, *M. sabuleti*, and *M. scabrinodis*) and from different populations of the same host species (*M. scabrinodis*).

## Material and methods

### Collection of ants

**Figure 2:**
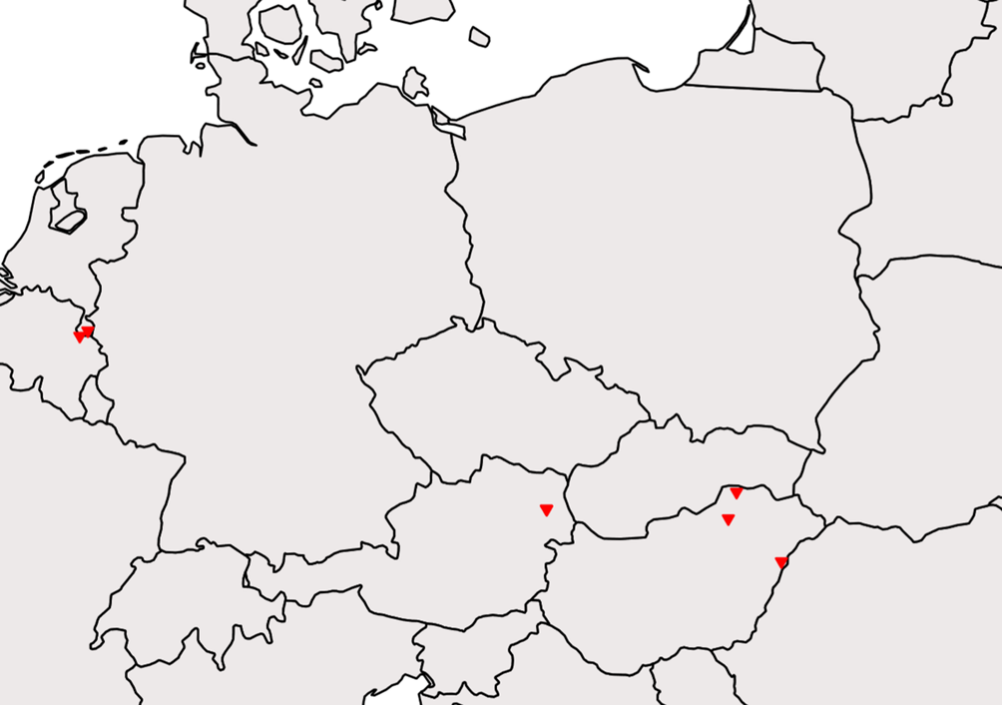
Field sites where ants for this project have been collected. Field sites are located in Europe (Austria, Belgium, Hungary, The Netherlands).

Ants were collected directly from nests in seven locations in four countries (Fig. 2): Austria (Vienna), Belgium (Moelingen), Hungary (Bükkszentkereszt, Rakaca, Újléta), and the Netherlands (Savelsbos, Wijlre-Eys). Long-term preservation was in 80–96% ethanol. Identification of ants was based on Seifert [64] and Radchenko and Elmes [56]. Voucher specimens are deposited at the Naturalis Biodiversity Center (Leiden, The Netherlands) and the Hungarian Natural History Museum (Budapest, Hungary). Identification of mounted thalli was done under light microscope, based on Thaxter [70] and De Kesel *et al*. [15].

### DNA extraction, PCR amplification, and sequencing

DNA was isolated from 3 to 100s of thalli using extraction protocols described in [31] or a modified REPLI-g Single Cell Kit (Qiagen, Valencia, California) [30]. The internal transcribed spacer (ITS) region (ITS1–5.8S–ITS2) and the 5′ end of the nuclear ribosomal large subunit (28S) were ampliﬁed, for ITS using primer pairs ITS1f [26] & ITS4 [77] and ITS9mun [19] & ITS4, for partial ITS–28S using the newly designed *Rickia*-specific primer RicITS2 (5′–CTAGTGTGAATTGCATATTTTAGTG–3′) & LR3 [74], and for 28S-only using LR0R [38] & LR5 [74] and NL1 & NL4 [44]. Polymerase chain reactions (PCR) used 13.3 μL of RedExtract Taq polymerase (Sigma–Aldrich, St. Louis, Missouri), 2.5 μL of each 10 μM primer, 5.7 μL of H_2_O, and 1.0 μL of template DNA. In some cases, 0.25 μL of dimethyl sulfoxide (DMSO) was added as a PCR enhancer (and 5.45 μL of H_2_O). All amplifications were done in an Applied Biosystems 2720 thermal cycler (Foster City, California) with initial denaturation at 94 °C for 3:00 min; followed by 35 cycles of denaturation at 94 °C for 1:00 min, annealing at 50 °C for 0:45 min, and extension at 72 °C for 1:30 min; and final extension at 72 °C for 10:00 min.

PCR products were loaded onto TAE 1% agarose gels for electrophoresis at 100 V for 25 min and UV transillumination was used to check the product size. Products showing strong bands on gel were purified with Qiaquick PCR Purification Kit (Qiagen) or DF100 PCR cleaning kit (Geneaid, New Taipei City, Taiwan) and sequenced using the same primers and 1 μL of purified PCR product per 10 μL sequencing reaction. Sequencing reactions were performed using the Big Dye^®^ Terminator v3.1 Cycle Sequencing Kit (Life Technologies, Carlsbad, California). For molecular work performed in Hungary, sequencing was outsourced to Microsynth AG (Wolfurt-Bahnhof, Austria). Sequence fragments were assembled, trimmed, and manually edited at ambiguous sites in Sequencher 4.10.1 (Gene Codes Corporation, Ann Arbor, Michigan). The identities of our consensus sequences were confirmed by performing BLAST searches at http://ncbi.nlm.nih.gov/blast/Blast.cgi. Edited sequences are deposited in NCBI GenBank (accession numbers in Table 1).

**Table 1:** Overview of *Rickia* sequences used in this study. All isolates for which sequences were generated are listed, with GenBank accession numbers as well as host species, country, and year of collection.

Isolate	Species	ITS	28S	Host	Country	Year
ADK6272a	*R. wasmannii*	MK500050	MK500050	*Myrmica sabuleti*	Belgium	2015
ADK6274c	*R. wasmannii*	MK500051	–	*Myrmica sabuleti*	Belgium	2015
DE_Rak4	*R. wasmannii*	KT800050	KT800021	*Myrmica scabrinodis*	Hungary	2014
Bükkszentkereszt2016	*R. wasmannii*	MK500052	–	*Myrmica scabrinodis*	Hungary	2016
Újléta2014	*R. wasmannii*	MK500053	MK490857	*Myrmica scabrinodis*	Hungary	2014
Újléta2015-4	*R. wasmannii*	MK500054	–	*Myrmica scabrinodis*	Hungary	2015
Wien2015-1	*R. wasmannii*	MK500055	MK490858	*Myrmica rubra*	Austria	2015
D. Haelew. 1234a	*R. wasmannii*	MH040595	MH040595	*Myrmica sabuleti*	Netherlands	2013
Wien2016-1	*R. wasmannii*	MK500056	–	*Myrmica rubra*	Austria	2016
Wiensabuleti2016-1	*R. wasmannii*	MK500057	–	*Myrmica sabuleti*	Austria	2016
SR1s	*R. pachyiuli*	MH040593	MH040593	*Pachyiulus hungaricus*	Serbia	2015
SR8s	*R. pachyiuli*	MK500058	MK500058	*Pachyiulus hungaricus*	Serbia	2015
SR13s	*R. pachyiuli*	MK500059	MK500059	*Pachyiulus hungaricus*	Serbia	2015
SR4s	*R. laboulbenioides*	MH040592	MH040592	*Cylindroiulus punctatus*	Denmark	2015
SR5s	*R. laboulbenioides*	MK500060	MK500060	*Cylindroiulus punctatus*	Denmark	2015
SR12s	*R. uncigeri*	MK500061	MK500061	*Unciger foetidus*	Denmark	2015

### Datasets and phylogenetic analyses

Individual datasets for ITS and 28S were constructed in order to assess intraspecific phylogenetic diversity in *Rickia wasmannii*. Alignments were done using MUSCLE v3.7 [18] on the Cipres Science Gateway v3.3 [50] and checked in BioEdit v7.2.6 [36]. Ambiguously aligned regions and uninformative positions were removed using trimAl v1.3 [8] with 60% gap threshold and minimal coverage of 50%. We also constructed a combined ITS + 28S dataset. The aligned sequence data for each region were concatenated in MEGA7 [43] to create a matrix of 804 bp with phylogenetic data for 16 isolates.

Maximum parsimony (MP) analyses were run using PAUP on XSEDE [69]. MP was estimated with heuristic searches consisting of 500 stepwise-addition trees obtained using random sequence addition replicates followed by tree bisection-reconnection (TBR) branch swapping (MulTrees in effect) and saving all equally most-parsimonious trees. Robustness of branches was estimated by maximum parsimony bootstrap proportions using 500 replicates, with heuristic searches consisting of 10 stepwise-addition trees obtained using random sequence addition replicates followed by TBR branch swapping, with MaxTrees set at 100. Maximum likelihood (ML) analyses were run using IQ-TREE [10, 51] from the command line. Nucleotide substitution models were selected under Akaike’s information criterion corrected for small sample size (AICc) with the help of jModelTest 2 [14] in Cipres [50]. For the ITS dataset, the TPM1 + G model was selected (−ln*L* = 712.0173); for 28S, the TrN + G model (−ln*L* = 1371.6022). ML was inferred for each individual dataset under the appropriate model, and for the concatenated dataset under partitioned models. Ultrafast bootstrap analysis was implemented with 1000 replicates [37]. Phylogenetic reconstructions with bootstrap values (BS) were visualised in FigTree v1.4.3 (http://tree.bio.ed.ac.uk/software/figtree/).

### Species delimitation

We used three species delimitation methods to validate species limits of or within *Rickia wasmannii* (*fide* [30, 34]): Automatic Barcode Gap Discovery method [55], General Mixed Yule Coalescent method [54], and a Poisson tree processes model approach [79]. All analyses were done with both the ITS and 28S datasets; the ITS region has been proposed as the universal barcode for all fungi [63] whereas the 28S locus was recently put forward as potential barcode for Laboulbeniomycetes because it is easy to amplify and has high discriminative power [30, 75]. We used the following parameters in the online version of ABGD (https://wwwabi.snv.jussieu.fr/public/abgd/abgdweb.html): *P*_min_ = 0.001, *P*_max_ = 0.01, steps = 10, *N*_b_ bins = 20. We evaluated results for both the Jukes-Cantor (JC69) and Kimura two-parameter (K80) distance metrics [40, 41] and for four gap width values (*X*): 0.1, 0.5, 1.0, and 1.5. We used the online version of bPTP (http://species.h-its.org) with default values for all parameters (number of MCMC generations, thinning, burn-in, seed). Finally, we conducted GMYC in R (R Core Team 2013) using the packages *rncl* [49] and *splits* [25]. The MCC tree from Bayesian inference (BI) served as input for both the bPTP and GMYC analyses.

Bayesian analyses were run for individual datasets with a Markov Chain Monte Carlo (MCMC) coalescent approach implemented in BEAST v1.8.4 [17], under a strict molecular clock assuming a constant rate of evolution across the tree. We selected the Birth-Death Incomplete Sampling speciation model [66] as tree prior and the nucleotide substitution model selected by jModelTest 2 [14] under AICc. Four independent runs were performed from a random starting tree for 10 million generations with a sampling frequency of 1000. Using the same settings failed to converge for the ITS dataset, and we thus optimised settings, selecting the GMRF Bayesian Skyride coalescent tree prior and increasing the number of generations to 80 million (with sampling frequency of 8000). Settings of priors were entered in BEAUti [17] to generate an XML file, which was run using BEAST on XSEDE in Cipres (two runs) and locally from the command line (two runs). The resulting log files were entered in Tracer [57] to check trace plots for convergence and to adjust burn-in. Burn-in values were changed for each log file to achieve net Effective Sample Sizes of ≥200 for sampled parameters. While removing a portion of each run as burn-in, log files and trees files were combined in LogCombiner. TreeAnnotator was used to generate consensus trees (0% burn-in) and to infer the Maximum Clade Credibility (MCC) tree, with the highest product of individual clade posterior probabilities.

### Thallus density counts

Thallus density was determined on 354 *Myrmica* workers. Workers originated from Austria (Vienna), Hungary (Bükkszentkereszt, Rakaca, Újléta), and the Netherlands (Savelsbos, Wijlre-Eys). Thalli of workers were counted under a stereomicroscope at 40×. Thalli were counted on workers of *M. rubra* (34 workers from Vienna), *M. sabuleti* (three workers from Savelsbos, 47 from Wijlre-Eys), and *M. scabrinodis* (50 workers from Bükkszentkereszt, 100 workers from Rakaca, 100 from Újléta, 20 from Wijlre-Eys). Counts were done on recently sampled workers. Counting took place with the workers submerged in H_2_O, which increased visibility of thalli.

### Statistical analyses

We used both absolute and relative values of counted thallus numbers for each body part in statistical analyses. The former is simply the number of thalli counted on a given body part, whereas the latter is calculated as the absolute number of thalli on a given body part divided by total number of thalli on the worker body. We used *R* for all presented statistical data analyses (R Core Team 2018).

#### Absolute and relative thallus numbers

To test for significant differences in total number of thalli between *Myrmica* species, we used a quasi-Poisson generalized linear regression model, in which the number of counted thalli was the response variable, and ant species was the predictor. Quasi-Poisson was preferred over a classical Poisson model, because the count-data showed considerable over-dispersion. Model summaries for models containing factor variables in *R* generally present parameter estimates contrasting them to an arbitrarily selected factor level, so with factor variables with more than two levels, some contrasts are not shown. To acquire factor level comparisons not shown in the summary, the package *lsmeans* was used [45].

Next, we compared absolute thallus number of given body parts between species. To do so, we used multiple Conover-Iman tests of the package *conover.test* [16]. In each test, we tested the difference between *Myrmica* species in the counted thallus number on a given body part. Following the tests (resulting in 48 comparisons), we applied Bonferroni’s *P*-value adjustment to avoid Type I error results. In the results, we only considered tests as significant if Bonferroni-adjusted *P*-values were below 0.05. We compared relative thallus number on given body parts as well, also using Conover-Iman tests. Similarly, we used Bonferroni’s adjustment on the *P*-values from the Conover-Iman test results.

To visualise species differences in the pattern of infection over the body of ants we used the Barnes-Hut implementation of t-distributed stochastic neighbour embedding (t-SNE [72, 73]) with the package *Rtsne* [42]. We chose this method over implementing a Principle Component Analysis approach, because in t-SNE we could explicitly specify the number of dimensions onto which to reduce the original data. Therefore, we were able to plot infection patterns (both of absolute and relative thallus number) on a 2D scatterplot. For t-SNE we used the square root-transformed values for both absolute and relative thallus numbers.

#### Potential origin of infection on the body

It has been suggested that infection with *R. wasmannii* starts from the ant head [15, 32, 48]. If so, one would expect to see that, in the early stages of infection, only (or mostly) the head is parasitised. Therefore, the relative number of thalli should be high during the first stages of infection (= when total number of thalli is small). Consequently, if the infection spreads from the head to other body parts, we should see a decrease in the relative thallus number on the head simultaneously with the increase of total number of thalli on the whole body.

First, we checked the range (minimum and maximum values) of the relative thallus number on each body part of infected ants, separately for the three ant species. We selected those body parts to be of interest in which one) the minimum value of relative thallus number was larger than zero and two) the maximum value was the largest in comparison to other body parts. In *M. rubra*, the frontal (or dorsal) side of the head and the gaster tergites satisfied our criteria. In *M. sabuleti*, only the frontal side of the head had a minimum relative thallus number value larger than zero, and it had the largest maximum value among all body parts. In *M. scabrinodis*, there was no body part on which the minimum value of relative thallus number was larger than zero, and so we selected the body part with the largest maximum value, which was the gaster tergites. Notably, on the gaster tergites of *M. scabrinodis*, we observed the lowest incidence of zero values in relative thallus number as well. As a result, we decided to use this body part as a starting point to test our hypothesis about the infection’s spread.

To test whether there is indeed a significant negative association between total thallus number and relative thallus number on selected body parts (frontal side of the head, gaster tergites), we used quasi-binomial generalised linear regression models. Using these, we were able to reliably fit models on a numeric scale ranging from zero to one (i.e. on the scale of the data) and to control for over-dispersion in the data. Two models were fitted. We specified the response variables to be the relative thallus number on the frontal side of the head and on the gaster tergites in the first and second model, respectively. In both models, predictor variables were total number of thalli on the whole body, a factor variable generated by specifying species name and country of origin, and the interaction term between these two variables. The factor variable had four levels: (1) *M. rubra* from Austria, (2) *M. sabuleti* from the Netherlands, (3) *M. scabrinodis* from Hungary, and (4) *M. scabrinodis* from the Netherlands.

In both models, we used square root-transformed response and predictor variables. Also, to be able to infer on mean species-level differences using the intercept estimates of the models, we centred the square root-transformed predictor variable at zero by subtracting the mean of the variable from each of its values. Furthermore, in the Results section we report actual regression coefficients for the slopes from each factor level, using the package *jtools* [47].

## Results

### Phylogenetic analyses, and species delimitation

**Figure 3:**
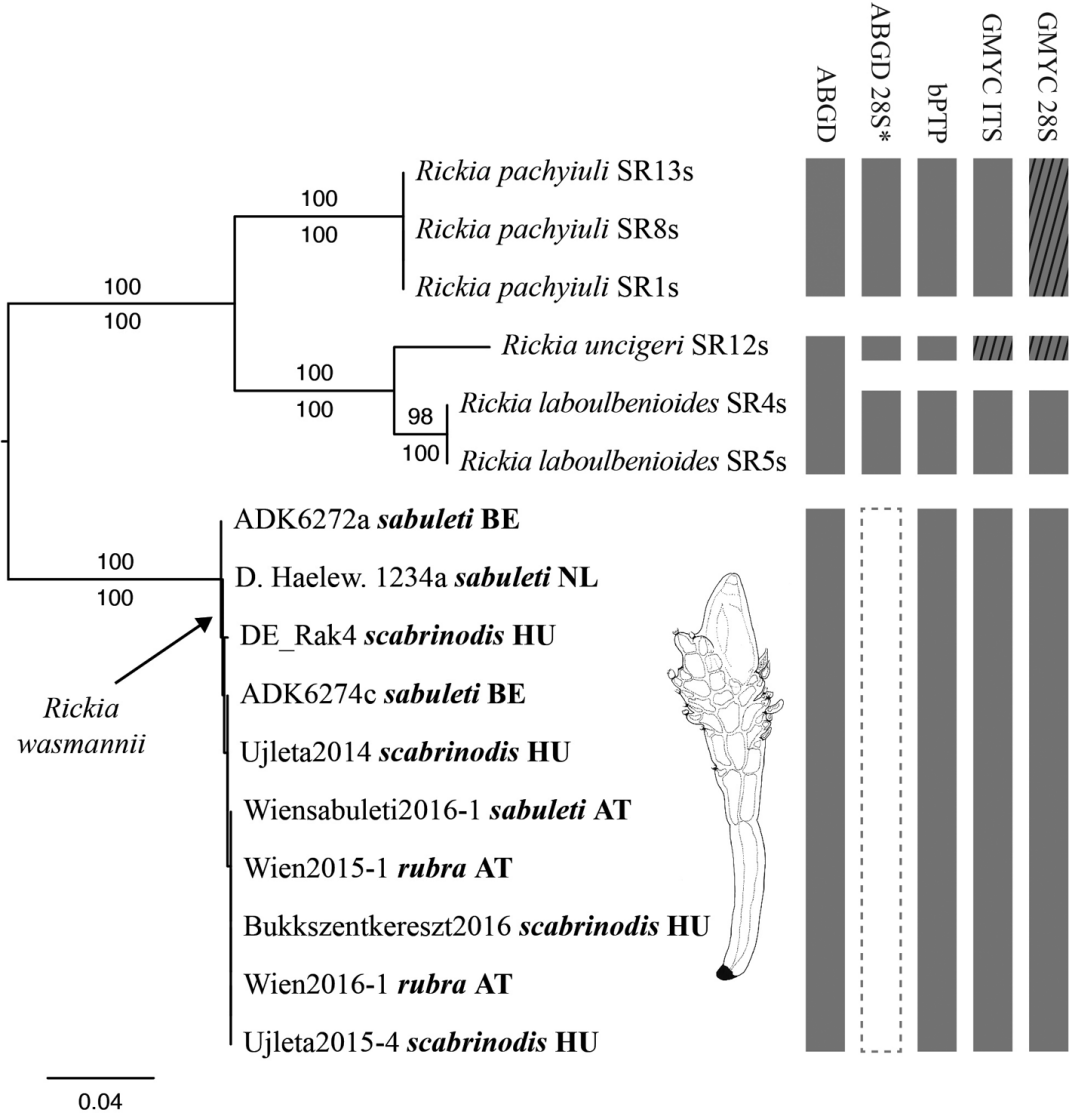
Phylogenetic reconstruction of *Rickia* species using a combined ITS + 28S dataset. The topology is the result of maximum likelihood inference. For each node, ML and MP bootstraps are presented above and below the branch leading to that node. For each *R. wasmannii* isolate, isolate name, *Myrmica* host epithet, and country code (AT, Austria; BE, Belgium; HU, Hungary; NL, The Netherlands) are presented. To the right of the phylogeny, results of species delimitation methods are summarised, from left to right: ABGD of the aligned ITS & 28S datasets under most parameters combinations; ABGD of the 28S dataset for *P* = 0.001–0.001668 and *X* = 0.1–1.0 (asterisk *); bPTP of the ITS and 28S topologies; and GMYC of the ITS and 28S ultrametric trees, respectively. Hatching implies lack of support, whereas the dashed rectangle under ABGD 28S* means that four putative species were found within *R. wasmannii*.

The ITS dataset comprised 258 characters, of which 182 were constant and 70 were parsimony-informative. A total of 16 isolates were included (Table 1): *Rickia wasmannii* (10 isolates as ingroup), *R. laboulbenioides* De Kesel (two isolates), *R. pachyiuli* M. Bechet & I. Bechet (three isolates), and *R. uncigeri* Scheloske (one isolate). *Rickia wasmannii* was retrieved as a monophyletic clade with maximum support from MP, ML, and BI (not shown). In this clade were included *R. wasmannii* isolates of thalli removed from *M. rubra* (two isolates), *M. sabuleti* (four isolates), and *M. scabrinodis* (four isolates). The 28S dataset comprised 547 characters, of which 423 were constant and 113 were parsimony-informative. A total of 11 isolates were included (Table 1): *Rickia wasmannii* (five isolates as ingroup), *R. laboulbenioides* (two isolates), *R. pachyiuli* (three isolates), and *R. uncigeri* (one isolate). *Rickia wasmannii* was retrieved as a monophyletic clade with maximum support from MP, ML, and BI (not shown), including isolates from *M. rubra* (one isolate), *M. sabuleti* (two isolates), and *M. scabrinodis* (two isolates). The concatenated ITS + 28S dataset comprised 804 characters, of which 604 were constant and 183 were parsimony-informative. A total of 16 isolates were included. Once again, *R. wasmannii* was retrieved as a monophyletic clade with maximum support from MP and ML (Fig. 3).

Results of the species delimitation methods are summarised in Tables 2 and 3 and Figure 3. The number of putative species of *Rickia* was three in the ITS dataset with ABGD: *Rickia laboulbenioides* + *uncigeri*, *R. pachyiuli*, and *R. wasmannii*. In the 28S dataset, this number varied from three to seven, depending on the prior intraspecific divergence parameter, whereas other parameters (relative gap width, distance metrics employed) had no influence on the results (Table 3). The bPTP analysis of both ITS and 28S topologies resulted in four highly supported species: *Rickia laboulbenioides*, *R. pachyiuli*, *R. uncigeri*, and *R. wasmannii*. The GMYC analysis of the ITS resulted in the recognition of four species, all with moderate to high support except for *R. uncigeri* (of which only a single isolate was included). The GMYC analysis of the 28S led to comparable results, but in this analysis also the *R. wasmannii* clade received low support (*pp* = 0.38).

**Table 2:** Summary of results of MP, ML, BI, and species delimitation analyses (ABGD, bPTP, GMYC). Explanation of symbols and values used: *pp* = posterior probability; + under ABGD represents supported clades; 4 under ABGD means that the analysis found support for 4 species hypotheses (*fide* [55]) within *R. wasmannii* under prior maximum distance (*P*) = 0.001, 0.001292, and 0.001668; numbers under bPTP and GMYC are Bayesian support values for delimited species hypotheses.

Putative species	MP BS	ML BS	*pp*	ABGD	ABGD	ABGD	bPTP	GMYC
				*P* = 0.001	*P* = 0.002783	*P* = 0.01		
ITS								
*R. wasmannii*	100	100	1.0	+	+	+	0.869	0.64
*R. laboulbenioides*	99.3	97	1.0	+	+	+	0.996	0.96
*R. uncigeri*	100	98	1.0				1.000	0.00
*R. pachyiuli*	99.8	98	1.0	+	+	+	0.962	0.80
28S								
*R. wasmannii*	100	100	1.0	4	+	+	0.974	0.38
*R. laboulbenioides*	99.9	97	1.0	+	+	+	0.994	0.76
*R. uncigeri*	99.8	98	1.0	+			1.000	0.00
*R. pachyiuli*	100	98	1.0	+	+	+	0.993	0.63

**Table 3:** Results of the Automatic Barcode Gap Discovery (ABGD) analyses. *X*, relative gap width; JC69, Jukes-Cantor substitution model; K80, Kimura 2-parameter substitution model.

		Prior intraspecific divergence (*P*)									
Distance	*X*	0.001	0.001292	0.001668	0.002154	0.002783	0.003594	0.004642	0.005995	0.007743	0.01
ITS											
JC69	0.1	3	3	3	3	3	3	3	3	3	3
	0.5	3	3	3	3	3	3	3	3	3	3
	1.0	3	3	3	3	3	3	3	3	3	3
	1.5	3	3	3	3	3	3	3	3	3	3
K80	0.1	3	3	3	3	3	3	3	3	3	3
	0.5	3	3	3	3	3	3	3	3	3	3
	1.0	3	3	3	3	3	3	3	3	3	3
	1.5	3	3	3	3	3	3	3	3	3	3
28S											
JC69	0.1	7	7	7	3	3	3	3	3	3	3
	0.5	7	7	7	3	3	3	3	3	3	3
	1.0	7	7	7	3	3	3	3	3	3	3
	1.5	3	3	3	3	3	3	3	3	3	3
K80	0.1	7	7	7	3	3	3	3	3	3	3
	0.5	7	7	7	3	3	3	3	3	3	3
	1.0	7	7	7	3	3	3	3	3	3	3
	1.5	3	3	3	3	3	3	3	3	3	3

### Absolute and relative thallus numbers

**Figure 4:**
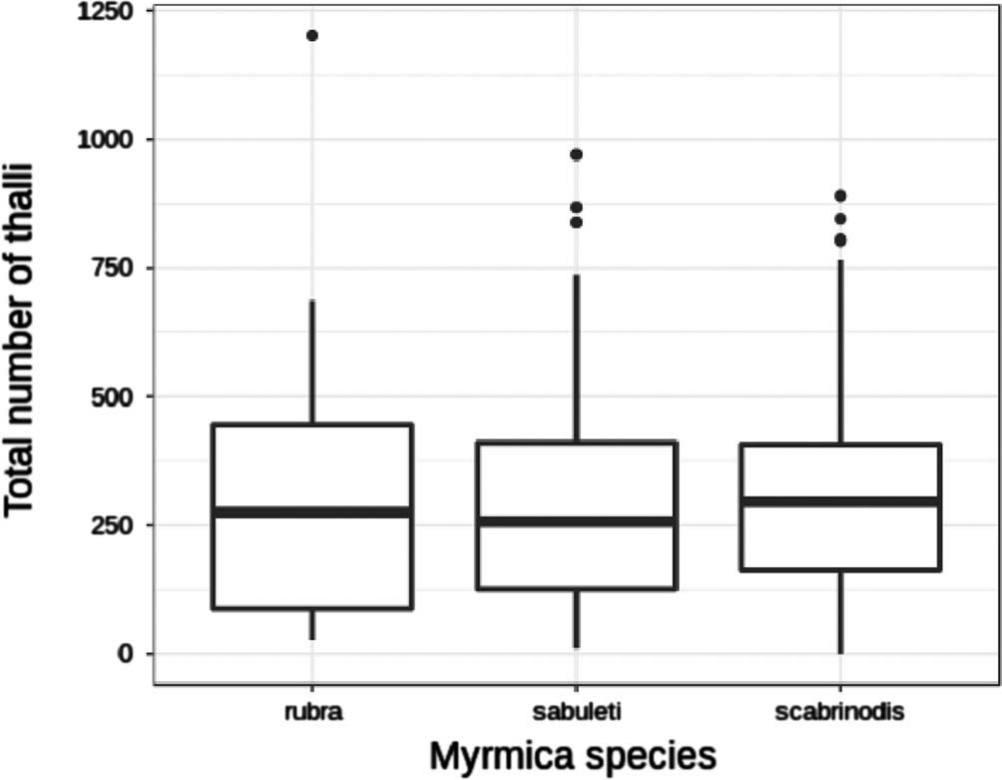
Total number of thalli on worker bodies of the three examined *Myrmica* species (*n* = 34 for *M. rubra*, *n* = 50 for *M. sabuleti*, *n* = 270 for *M. scabrinodis*).

**Figure 5:**
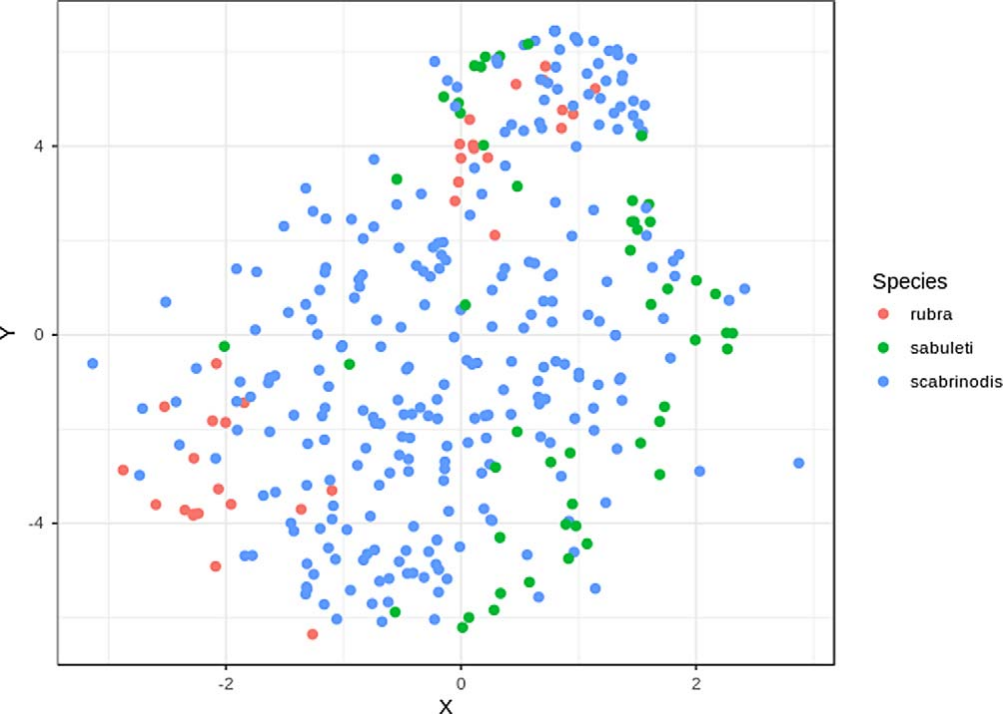
Visualisation of infection patterns based on absolute thallus number on 16 body parts, using t-SNE to reduce the number of dimensions of the data set (*n* = 354).

There were no significant differences between *Myrmica* species in total thallus number (Table 4, Fig. 4). In the Conover-Iman tests comparing absolute thallus number of each body part between species, we found 20 significant differences after Bonferroni’s *P*-value adjustment (Table 5). Overall, *M. sabuleti* specimens were more heavily infected on their antennae, head, pronotum, and mesonotum compared to workers of *M. rubra* and *M. scabrinodis*, whereas *M. scabrinodis* ants appeared to have the highest thallus density on their petiole and gaster tergites in comparison to the other host species. *Myrmica rubra* workers showed highest thallus density on their gaster sternites, procoxa, mesocoxa, and metacoxa (Fig. 5).

**Table 4:** Contrasts acquired from the model estimating between-species differences in total thalli number.

Contrasts of *Myrmica* spp.	Estimate	*SE*	*z*-ratio	*P*
*M. rubra – M. sabuleti*	−0.070	0.155	−0.45	0.893
*M. rubra – M. scabrinodis*	−0.046	0.128	−0.36	0.932
*M. sabuleti – M. scabrinodis*	0.024	0.105	0.23	0.971

**Table 5:** Results of Conover-Iman tests on the species differences of body parts in absolute thallus density. The column “Largest thallus density” shows which host species had highest number of thalli on a given body part; species names in parentheses indicate that the difference between the specified species and one of the other two species is not significant; “none” means that the three species did not differ significantly from one another.

Body part	Comparisons of *Myrmica* spp.	*t*	*P* (adjusted)	Largest thallus density
Antennae	*M. rubra* – *M. sabuleti*	−5.28	<0.001	*M. sabuleti*
	*M. rubra* – *M. scabrinodis*	−1.56	1.000	
	*M. sabuleti* – *M. scabrinodis*	5.78	<0.001	
Head (frontal)	*M. rubra* – *M. sabuleti*	−3.15	0.029	(*M. sabuleti*)
	*M. rubra* – *M. scabrinodis*	−3.09	0.034	
	*M. sabuleti* – *M. scabrinodis*	0.90	1.000	
Head (ventral)	*M. rubra* – *M. sabuleti*	−0.52	1.000	(*M. sabuleti*)
	*M. rubra* – *M. scabrinodis*	2.26	0.254	
	*M. sabuleti* – *M. scabrinodis*	3.43	0.012	
Pronotum	*M. rubra* – *M. sabuleti*	−4.54	<0.001	*M. sabuleti*
	*M. rubra* – *M. scabrinodis*	−2.96	0.048	
	*M. sabuleti* – *M. scabrinodis*	3.06	0.036	
Mesonotum	*M. rubra* – *M. sabuleti*	−4.64	<0.001	(*M. sabuleti*)
	*M. rubra* – *M. scabrinodis*	−3.48	0.011	
	*M. sabuleti* – *M. scabrinodis*	2.59	0.127	
Propodeum	*M. rubra* – *M. sabuleti*	−2.65	0.110	None
	*M. rubra* – *M. scabrinodis*	−2.80	0.076	
	*M. sabuleti* – *M. scabrinodis*	0.52	1.000	
Petiole	*M. rubra* – *M. sabuleti*	−3.29	0.018	(*M. scabrinodis*)
	*M. rubra* – *M. scabrinodis*	−4.05	0.001	
	*M. sabuleti* – *M. scabrinodis*	−0.03	1.000	
Postpetiole	*M. rubra* – *M. sabuleti*	−2.46	0.173	None
	*M. rubra* – *M. scabrinodis*	−2.30	0.242	
	*M. sabuleti* – *M. scabrinodis*	0.83	1.000	
Gaster tergites	*M. rubra* – *M. sabuleti*	0.96	1.000	(*M. scabrinodis*)
	*M. rubra* – *M. scabrinodis*	−2.35	0.224	
	*M. sabuleti* – *M. scabrinodis*	−4.17	0.001	
Gaster sternites	*M. rubra* – *M. sabuleti*	3.48	0.011	(*M. rubra*)
	*M. rubra* – *M. scabrinodis*	0.07	1.000	
	*M. sabuleti* – *M. scabrinodis*	−4.95	<0.001	
Procoxa	*M. rubra* – *M. sabuleti*	3.39	0.014	*M. rubra*
	*M. rubra* – *M. scabrinodis*	4.74	<0.001	
	*M. sabuleti* – *M. scabrinodis*	0.70	1.000	
Profemur	*M. rubra* – *M. sabuleti*	1.22	1.000	None
	*M. rubra* – *M. scabrinodis*	0.60	1.000	
	*M. sabuleti* – *M. scabrinodis*	−1.06	1.000	
Mesocoxa	*M. rubra* – *M. sabuleti*	2.67	0.107	(*M. rubra*)
	*M. rubra* – *M. scabrinodis*	3.80	0.003	
	*M. sabuleti* – *M. scabrinodis*	0.64	1.000	
Mesofemur	*M. rubra* – *M. sabuleti*	0.25	1.000	None
	*M. rubra* – *M. scabrinodis*	1.53	1.000	
	*M. sabuleti* – *M. scabrinodis*	1.44	1.000	
Metacoxa	*M. rubra* – *M. sabuleti*	3.30	0.018	*M. rubra*
	*M. rubra* – *M. scabrinodis*	3.79	0.003	
	*M. sabuleti* – *M. scabrinodis*	−0.29	1.000	
Metafemur	*M. rubra* – *M. sabuleti*	1.48	1.000	None
	*M. rubra* – *M. scabrinodis*	1.28	1.000	
	*M. sabuleti* – *M. scabrinodis*	−0.63	1.000	

**Figure 6:**
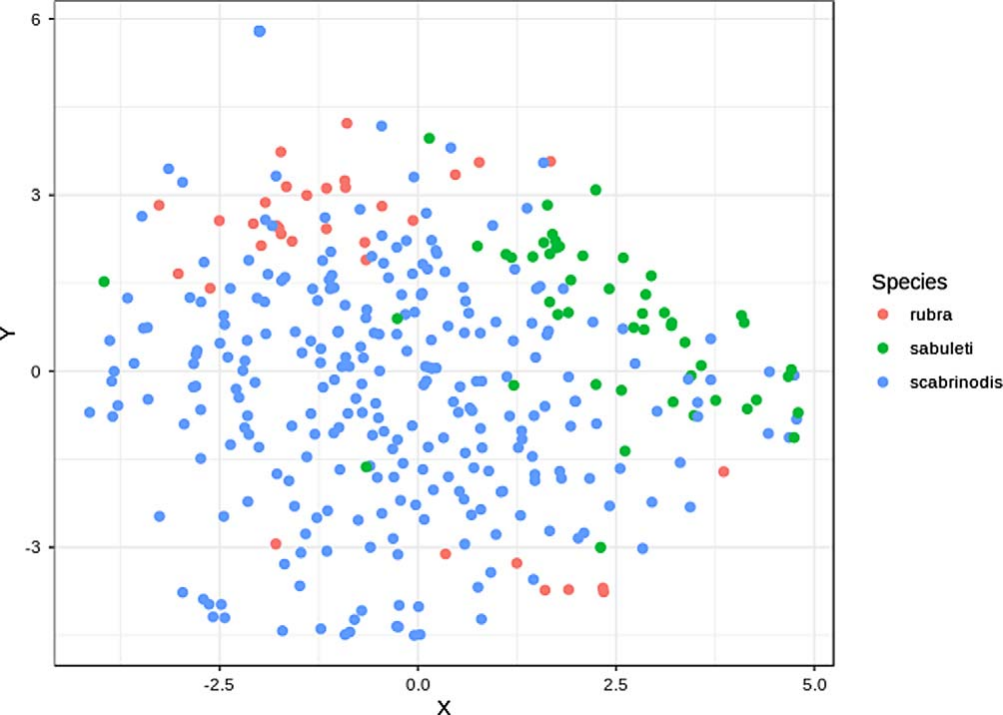
Visualisation of infection patterns based on relative thallus number on 16 body parts, using t-SNE to reduce the number of dimensions of the data set (*n* = 354).

In the Conover-Iman tests comparing relative thallus number of each body part between species, we found 29 significant differences after Bonferroni’s *P*-value adjustment (Table 6). In comparison to *M. rubra* and *M. scabrinodis*, values of relative thallus number were highest in *M. sabuleti* on the antennae, head, pronotum, mesonotum, propodeum, and petiole. On the gaster tergites, *M. scabrinodis* had larger proportions of thalli compared to the other host species. Furthermore, *M. rubra* workers had highest proportions of thalli on their gaster sternites, procoxa, mesocoxa, mesofemur, metacoxa, and metafemur, compared to the other host species (Fig. 6).

**Table 6:** Results of Conover-Iman tests on the species differences of body parts in relative thallus number. The column “Largest proportion of thalli” shows which host species had highest proportion of thalli on a given body part; species names in parentheses indicate that the difference between the specified species and one of the other two species is not significant; “none” means that the three species did not differ significantly from one another.

Body part	Comparisons of *Myrmica* spp.	*t*	*P* (adjusted)	Largest proportion of thalli
Antennae	*M. rubra* – *M. sabuleti*	−6.77	<0.001	*M. sabuleti*
	*M. rubra* – *M. scabrinodis*	−1.77	0.542	
	*M. sabuleti* – *M. scabrinodis*	7.68	<0.001	
Head (frontal)	*M. rubra* – *M. sabuleti*	−3.59	0.005	*M. sabuleti*
	*M. rubra* – *M. scabrinodis*	−1.68	0.616	
	*M. sabuleti* – *M. scabrinodis*	3.20	0.017	
Head (ventral)	*M. rubra* – *M. sabuleti*	−0.15	1.000	(*M. sabuleti*)
	*M. rubra* – *M. scabrinodis*	4.49	<0.001	
	*M. sabuleti* – *M. scabrinodis*	5.53	<0.001	
Pronotum	*M. rubra* – *M. sabuleti*	−7.40	<0.001	*M. sabuleti*
	*M. rubra* – *M. scabrinodis*	−4.04	0.001	
	*M. sabuleti* – *M. scabrinodis*	5.91	<0.001	
Mesonotum	*M. rubra* – *M. sabuleti*	−7.33	<0.001	*M. sabuleti*
	*M. rubra* – *M. scabrinodis*	−4.36	<0.001	
	*M. sabuleti* – *M. scabrinodis*	5.43	<0.001	
Propodeum	*M. rubra* – *M. sabuleti*	−4.23	<0.001	(*M. sabuleti*)
	*M. rubra* – *M. scabrinodis*	−3.29	0.013	
	*M. sabuleti* – *M. scabrinodis*	2.22	0.231	
Petiole	*M. rubra* – *M. sabuleti*	−4.03	0.001	*M. sabuleti*
	*M. rubra* – *M. scabrinodis*	−4.94	<0.001	
	*M. sabuleti* – *M. scabrinodis*	−0.03	1.000	
Postpetiole	*M. rubra* – *M. sabuleti*	−2.00	0.368	None
	*M. rubra* – *M. scabrinodis*	−1.32	0.934	
	*M. sabuleti* – *M. scabrinodis*	1.34	0.934	
Gaster tergites	*M. rubra* – *M. sabuleti*	3.73	0.003	(*M. scabrinodis*)
	*M. rubra* – *M. scabrinodis*	−2.38	0.160	
	*M. sabuleti* – *M. scabrinodis*	−8.20	<0.001	
Gaster sternites	*M. rubra* – *M. sabuleti*	5.26	<0.001	(*M. rubra*)
	*M. rubra* – *M. scabrinodis*	0.58	1.000	
	*M. sabuleti* – *M. scabrinodis*	−6.90	<0.001	
Procoxa	*M. rubra* – *M. sabuleti*	5.11	<0.001	*M. rubra*
	*M. rubra* – *M. scabrinodis*	7.41	<0.001	
	*M. sabuleti* – *M. scabrinodis*	1.38	0.934	
Profemur	*M. rubra* – *M. sabuleti*	1.85	0.489	None
	*M. rubra* – *M. scabrinodis*	1.64	0.616	
	*M. sabuleti* – *M. scabrinodis*	−0.73	1.000	
Mesocoxa	*M. rubra* – *M. sabuleti*	3.49	0.007	*M. rubra*
	*M. rubra* – *M. scabrinodis*	5.00	<0.001	
	*M. sabuleti* – *M. scabrinodis*	0.88	1.000	
Mesofemur	*M. rubra* – *M. sabuleti*	0.80	1.000	(*M. rubra*)
	*M. rubra* – *M. scabrinodis*	3.01	0.028	
	*M. sabuleti* – *M. scabrinodis*	2.41	0.155	
Metacoxa	*M. rubra* – *M. sabuleti*	4.18	0.001	*M. rubra*
	*M. rubra* – *M. scabrinodis*	5.34	<0.001	
	*M. sabuleti* – *M. scabrinodis*	0.27	1.000	
Metafemur	*M. rubra* – *M. sabuleti*	3.07	0.025	*M. rubra*
	*M. rubra* – *M. scabrinodis*	3.17	0.018	
	*M. sabuleti* – *M. scabrinodis*	−0.68	1.000	

### Potential origin of infection on the body

**Figure 7:**
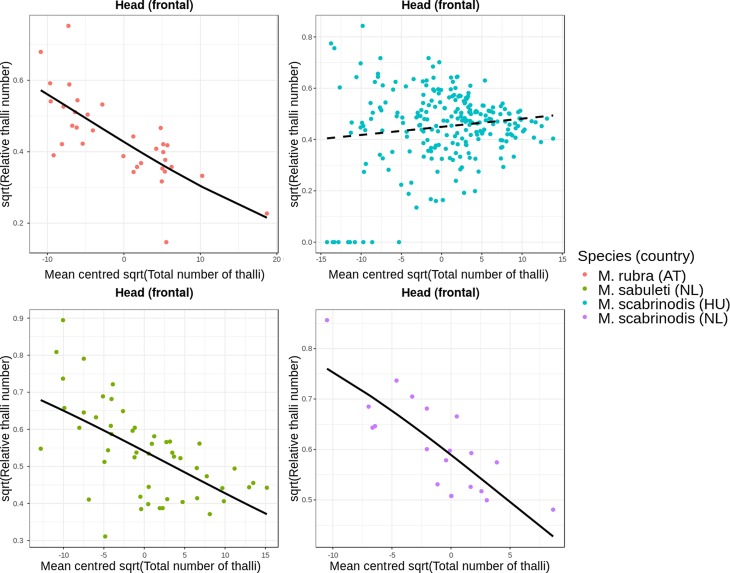
Association between total number of thalli and relative number of thalli on the frontal side of the head (*n* = 354). Black curves are regression lines from the fitted model: solid and dashed lines represent significant and non-significant associations, respectively.

The total number of thalli was significantly negatively associated with relative thallus number on the frontal side of the head in *M. rubra* (Estimate = −0.054, *SE* = 0.014, *t* = −3.81, *P* < 0.001) and in *M. sabuleti* (Estimate = −0.046, *SE* = 0.011, *t* = −3.97, *P* < 0.001). In the case of *M. scabrinodis*, the regression coefficients differed between ants from the Netherlands and Hungary: in the Netherlands, the association between total number of thalli and relative thallus number on the frontal side of the head was negative and relatively strong (Estimate = −0.076, *SE* = 0.029, *t* = −2.57, *P* = 0.011), whereas in Hungary, the regression coefficient was positive and weaker than in the other groups (Estimate = 0.013, *SE* = 0.006, *t* = 2.27, *P* = 0.024). These associations are shown in Figure 7.

**Figure 8:**
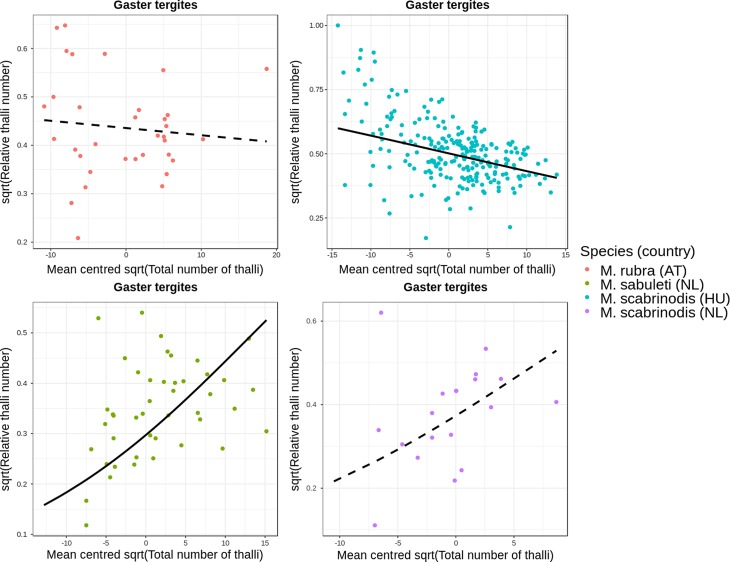
Association between total number of thalli and relative number of thalli on the gaster tergites (zeros excluded, *n* = 336). Black curves are regression lines from the fitted model: solid and dashed lines represent significant and non-significant associations, respectively.

In the model in which we fitted the relative thallus number of gaster tergites on total thallus number, the regression coefficient was significantly negative for *M. scabrinodis* ants collected in Hungary (Estimate = −0.042, *SE* = 0.005, *t* = −9.02, *P* < 0.001), but it was not significant in workers from the Netherlands (Estimate = 0.039, *SE* = 0.026, *t* = 1.52, *P* = 0.129). The association was not significant in *M. rubra* either (Estimate = −0.006, *SE* = 0.010, *t* = −2.57, *P* = 0.011). However, we found a significantly positive effect in *M. sabuleti* (Estimate = 0.024, *SE* = 0.011, *t* = 2.16, *P* = 0.031). These associations are shown in Figure 8.

## Discussion

*Rickia wasmannii* was described in the 19th century, based on morphological characters only [9]. *Myrmica* hosts of *R. wasmannii* belong in two phylogenetically distinct species groups (*rubra*-group and *scabrinodis*-group). The genus *Myrmica* quickly diversified around the Eocene–Oligocene transition. The *scabrinodis*-group is among the oldest species groups (21.46 ± 4.00 Mya), whereas the estimated crown age for the *rubra*-group is 10.88 ± 2.12 Mya, in the Late Miocene [39]. Our results demonstrate that *R. wasmannii* does not encompass divergent genetic lineages segregated by host. In all molecular phylogenetic reconstructions, *R. wasmannii* isolates formed a monophyletic clade with maximum support. Infected workers of *Myrmica* spp. were collected in Austria, Belgium, Hungary, and the Netherlands. Even so, there is no geographic signal. The ITS sequences of *R. wasmannii* are all identical, whereas there are 0, 1, or 2 nucleotide differences among LSU sequences. We conclude that, contrary to species of *Gloeandromyces* and *Hesperomyces*, in *R. wasmannii* neither geography nor host species are drivers of divergent evolution. The absence of host specificity in *R. wasmannii* is quite different from what has been observed in a *Myrmica*-associated group of endosymbiotic bacteria; *Spiroplasma* species co-diverged with their hosts over evolutionary time [1].

Most species of Laboulbeniales have been described based on morphological characters. In fact, only four species and four *formae* have been described based on combined morphological and molecular data [27, 34, 35]. In addition, for only a handful of species the taxonomic status has been assessed using molecular phylogenetic data following description. For example, *Corethromyces bicolor* Thaxt., after having been transferred to another genus, was re-installed in the genus *Corethromyces* Thaxt. based on DNA studies [76], and using sequence data from three loci, distinct clades within *Hesperomyces virescens* were found, each corresponding to host-speciﬁc species [30].

Species in the genus *Coreomyces* Thaxt. do not show host specificity – similar to *R. wasmannii*. *Coreomyces corixae* (green clade in [68]), for example, occurs on water boatmen (Heteroptera, Corixidae) in the genera *Callicorixa* White 1873, *Hesperocorixa* Kirkaldy 1908, and *Sigara* Fabricius, 1775 [67]. As more examples of Laboulbeniales fungi are explored, we can start linking speciation patterns to presence and absence of fungal traits. One candidate trait that may have an influence in host-dependent speciation in Laboulbeniales is the presence of a haustorium. Haustoria are rhizoidal structures that can be simple or branched and penetrate the host’s integument to provide additional holdfast and to increase surface area, presumably for nutrient uptake. Benjamin [6] believed that all Laboulbeniales produce haustoria. This is contrary to Tragust *et al*. [71] who, based on light and electron microscopy techniques, found no evidence for penetration in four species of Laboulbeniales: *Laboulbenia camponoti* S.W.T. Batra, *L. formicarum* Thaxt., *Rickia lenoirii* Santam., and *R. wasmannii*.

### A note about millipede-associated Laboulbeniales

The sequences of *R. laboulbenioides*, *R. pachyiuli*, and *R. uncigeri* were generated for this study and are the first published ones for millipede-associated Laboulbeniales. Laboulbeniales on millipedes occur in five genera: *Diplopodomyces* W. Rossi & Balazuc, *Rickia*, the recently described *Thaxterimyces* Santam., Reboleira & Enghoff, *Triainomyces* W. Rossi & A. Weir, and *Troglomyces* S. Colla [15, 22, 58–61]. Similar to our findings with bat fly-associated Laboulbeniales fungi [33], we expect that parasitism of millipedes by Laboulbeniales arose several times independently. Some species of *Rickia* on millipedes are known to parasitise several millipede hosts. For example, *R. candelabriformis* Santam., Enghoff & Reboleira*, R. gigas* Santam., Enghoff & Reboleira, and *R. lophophora* Santam., Enghoff & Reboleira [60] are potential next targets to study intraspecific diversity, to assess whether our current observations for *R. wasmannii* hold for the entire genus.

### Species delimitation analyses

The ABGD analysis of the 28S dataset found different numbers of putative species depending on the prior intraspecific divergence (*P*), which is in line with previous work. Puillandre *et al*. [55] put forward to use *P* = 0.01, because under this setting, ABGD results in the same number of putative species found using different approaches. In our analyses of both the ITS and 28S datasets, ABGD found three species under this setting: *R. laboulbenioides*+*uncigeri*, *R. pachyiuli*, and *R. wasmannii*. Checking the distance matrices, we found that the lowest number of inter-species nucleotide differences was observed between *R. laboulbenioides* and *R. uncigeri* (Tables 7 and 8). For example, in the ITS dataset, *R. laboulbenioides* differed in six nucleotides from *R. uncigeri*, whereas it differed in 16 nucleotides from *R. pachyiuli* and in 24 nucleotides from *R. wasmannii* (details in Table 7). Apparently, ABGD was not able to identify the divergence among *R. laboulbenioides* and *R. uncigeri* isolates as a “barcode gap” (*fide* [55]), which will likely be resolved once we generate and include more sequences of *R. uncigeri*. The GMYC results are congruent with the results from the other species delimitation methods. One clade lacks support, the singleton clade *R. uncigeri*, and this is no surprise because GMYC looks at intraspecific branching versus interspecific branching.

**Table 7:** Distance matrix of the aligned ITS sequences.

	Isolate	Species	GenBank acc. no.	1	2	3	4	5	6	7	8	9	10	11	12	13	14	15	16
1	SR4s	*Rickia laboulbenioides*	MH040592																
2	SR5s	*Rickia laboulbenioides*	MK500060	0															
3	SR8s	*Rickia pachyiuli*	MK500058	16	16														
4	SR1s	*Rickia pachyiuli*	MH040593	16	16	0													
5	SR13s	*Rickia pachyiuli*	MK500059	16	16	0	0												
6	SR12s	*Rickia uncigeri*	MK500061	6	6	20	20	20											
7	Újléta2014	*Rickia wasmannii*	MK500053	24	24	25	25	25	25										
8	DE_Rak4	*Rickia wasmannii*	KT800050	24	24	25	25	25	25	0									
9	Wien2015-1	*Rickia wasmannii*	MK500055	24	24	25	25	25	25	0	0								
10	D. Haelew. 1234a	*Rickia wasmannii*	MH040595	24	24	25	25	25	25	0	0	0							
11	ADK6272a	*Rickia wasmannii*	MK500050	24	24	25	25	25	25	0	0	0	0						
12	ADK6274c	*Rickia wasmannii*	MK500051	24	24	25	25	25	25	0	0	0	0	0					
13	Wien2016-1	*Rickia wasmannii*	MK500056	24	24	25	25	25	25	0	0	0	0	0	0				
14	Wiensabuleti2016-1	*Rickia wasmannii*	MK500057	24	24	25	25	25	25	0	0	0	0	0	0	0			
15	Bükkszentkereszt2016	*Rickia wasmannii*	MK500052	24	24	25	25	25	25	0	0	0	0	0	0	0	0		
16	Újléta2015-4	*Rickia wasmannii*	MK500054	24	24	25	25	25	25	0	0	0	0	0	0	0	0	0	

**Table 8:** Distance matrix of the aligned 28S rDNA sequences.

	Isolate	Species	GenBank acc. no.	1	2	3	4	5	6	7	8	9	10	11
1	SR4s	*Rickia laboulbenioides*	MH040592											
2	SR5s	*Rickia laboulbenioides*	MK500060	0										
3	SR8s	*Rickia pachyiuli*	MK500058	39	39									
4	SR1s	*Rickia pachyiuli*	MH040593	39	39	0								
5	SR13s	*Rickia pachyiuli*	MK500059	39	39	0	0							
6	SR12s	*Rickia uncigeri*	MK500061	19	19	42	42	42						
7	Újléta2014	*Rickia wasmannii*	MK490857	79	79	67	67	67	76					
8	DE_Rak4	*Rickia wasmannii*	KT800021	79	79	68	68	68	77	1				
9	Wien2015-1	*Rickia wasmannii*	MK490858	80	80	68	68	68	77	1	2			
10	D. Haelew. 1234a	*Rickia wasmannii*	MH040595	79	79	67	67	67	76	0	1	1		
11	ADK6272a	*Rickia wasmannii*	MK500050	79	79	67	67	67	76	0	1	1	0	

The lack of phylogenetic structuring among *R. wasmannii* populations may be attributed to two different but not mutually exclusive scenarios: (1) intermittent gene flow homogenising populations and (2) recent spread of the fungus starting from a small founder population. The first scenario is possible because co-occurring arthropods may share Laboulbeniales parasites. Interspecific ascospore transmission in sympatric species has been observed for *R. wasmannii* parasitising *M. scabrinodis*, mites, and a *Microdon myrmicae* larva (Diptera, Syrphidae) in ant nests [53]. The second scenario can best be illustrated with the following example. *Laboulbenia formicarum* Thaxt. is thought to have spread from North America to Europe on an unknown ant host [23], followed by host shifts to European-native and invasive ant host species during its rapid spread in recent years [24, 28]. It was shown for *M. rubra* that it survived the last glacial period in multiple refugia and expanded its distribution along different routes [46]. It might be possible that *R. wasmannii* has undergone postglacial spread with its host, followed by multiple host shifts to other *Myrmica* species. Microsatellite studies are required to assess population-wide genetic differences, e.g., to answer the question whether incipient sympatric speciation is taking place.

### Habitat specificity and host spectrum

*Rickia wasmannii* is a single species, clearly shared by a number of *Myrmica* hosts and with a vast distribution area. The species is non-penetrating [71] and compared to taxa with a haustorium such as *H. virescens*, it has several hosts but only if these occupy a similar habitat (*Myrmica* nests). This habitat specificity – preference for *Myrmica* nests and habitat choices – can explain the wide distribution on multiple *Myrmica* species and ant nest inquilines [53]. Moreover, the fact that there is overlap and even contact between *Myrmica* populations of different species [78] implies that regular or at least sufficient interspecific transfer of *R. wasmannii* occurs between host taxa. It also means that the different host taxa, their specific habitat choices, and the nature of their nests, allow the development of the fungus population. Considering the high thallus densities observed, we doubt there is enough reason to consider one *Myrmica* species as a main host (*fide* [62]) over other species. In this context, we propose that *R. wasmannii* is a true eurytopic species with a wide ecological amplitude. It is expected that other species of *Myrmica* may also carry this parasite. However, absence of *R. wasmannii* on a given *Myrmica* species does not necessarily mean that this ant species, its nests, and/or its habitat selection are unsuitable for this fungus. Indeed, in areas where several infected nests of *M. scabrinodis* occur, some adjacent nests can be entirely free of *R. wasmannii* [15]. This has also been observed for *M. sabuleti*, where infection frequency of workers can vary from 0 to 100% among nests that are only a few meters apart (P. Boer, unpublished data).

### Distribution of thalli on worker bodies

The original morphological description [8] holds to the phylogenetic species concept. This implies that differences in thallus numbers of different body parts between ant species and populations must be explained by behaviour, cuticular chemical profiles, and/or environmental stresses [7, 20, 21, 65]. In our dataset, there was no evidence for differences between host species in total number of thalli on worker bodies. If the ant species in the study area are of the same body size, this might suggest that the overall number of thalli on a worker’s body is simply a factor of the worker’s age, irrespective of host species; older workers show heavier infection by *R. wasmannii* [3].

We did observe differences between *Myrmica* species in the pattern of infection over the body. Tests on both absolute and relative thallus number indicate that *M. sabuleti* workers are more heavily infected on the first few body segments compared to other hosts (Tables 5 and 6). *Myrmica rubra* workers show highest thallus densities on the coxa and femur, whereas in *M. scabrinodis* highest thallus densities were found on the gaster tergites. These results might indicate differences among host species in how ants come into contact with the fungus, or even differences in grooming efficacy. Which body parts are workers able to groom (and thus stop ascospores from adhering and developing) more effectively? Another possibility involves differences in the cuticle itself [20, 65]; surface characteristics may have a fundamental impact on the success of an ascospore to adhere to the cuticle and develop to a mature thallus.

Based on our statistical analyses, it is likely that in *M. rubra* and *M. sabuleti* the locus of infection (= the area where the infection originates) is the frontal side of the head. For *M. scabrinodis* workers from the Netherlands, the locus of infection also appears to be the frontal side of the head. However, for *M. scabrinodis* specimens from Hungary, infection likely starts from the gaster tergites. These results indicate differences among populations of the same species in a wide geographical range. Different *Myrmica* species display divergent foraging, allo-grooming, and secretion emission activities [7]. This likely leads to differences in how workers enter into contact with ascospores, which should be investigated with behavioural studies of their hosts.
